# Self-perceived care needs in older adults with joint pain and comorbidity

**DOI:** 10.1007/s40520-017-0795-7

**Published:** 2017-07-07

**Authors:** Lotte A. H. Hermsen, Emiel O. Hoogendijk, Johannes C. van der Wouden, Martin Smalbrugge, Stephanie S. Leone, Henriëtte E. van der Horst, Joost Dekker

**Affiliations:** 1 Department of General Practice and Elderly Care Medicine, Amsterdam Public Health research institute, VU University Medical Center, Amsterdam, The Netherlands; 2 Department of Epidemiology and Biostatistics, Amsterdam Public Health research institute, VU University Medical Center, Amsterdam, The Netherlands; 3 Public Mental Health, Netherlands Institute of Mental Health and Addiction, Utrecht, The Netherlands; 4 Department of Rehabilitation Medicine, VU University Medical Center, Amsterdam, The Netherlands; 5 Department of Psychiatry, VU University Medical Center, Amsterdam, The Netherlands

**Keywords:** Joint pain, Comorbidity, Older adults, Primary care, Care needs, CANE

## Abstract

**Background:**

The aim of this study was to explore self-perceived care needs and determinants of identified needs in older adults with joint pain and comorbidity.

**Methods:**

This is a cross-sectional study using baseline data from a cohort study of older adults in the Netherlands (≥65 years) with joint pain and comorbidity (*n* = 407). We used the Camberwell Assessment of Need for the Elderly (CANE) to assess self-perceived care needs. Regression analyses were conducted to examine the associations between needs and sociodemographic factors (age, gender, partner status and educational level), physical factors (pain intensity, comorbidity, frailty and physical functioning) and psychosocial factors (anxiety, depression and social support).

**Results:**

Older adults with joint pain and comorbidity reported on average 4.0 care needs out of 13 CANE items, of which 0.3 were unmet. High levels of environmental and physical needs were reported, such as needs with regard to physical illness (91%), household (61%) and mobility/falls (53%). However, most of these needs were met. Only few people reported psychosocial needs, but a large proportion of these needs was unmet, especially regarding company (66.7%) and daytime activities (37%). Psychosocial needs were more often present in frail participants (OR 2.40, 95% CI 1.25–4.61), and those with less perceived social support (OR 1.05, 95% CI 1.01–1.08) and more depressive symptoms (OR 1.17, 95% CI 1.07–1.26).

**Discussion/Conclusions:**

Unmet needs are mainly present in the psychosocial domain. Specific attention targeted at these unmet needs may improve psychosocial well-being of older adults with joint pain and comorbidity.

## Introduction

In later life, joint pain is a common complaint. Previous research showed that 68–85% of older adults with joint pain also have at least one other chronic disease (comorbidity), like diabetes or ischemic heart disease [1, 2]. The distinct consequences of joint pain and those of several other health conditions such as disability are well documented [3, 4]. However, less is known about the impact of joint pain, in the presence of comorbidity, on levels of functioning and subsequent care needs of this specific population. Identifying care needs is regarded as an essential first step in optimizing health care, as it facilitates the implementation of early treatment strategies that aim to improve health, physical functioning and quality of life and subsequently can prevent or delay deterioration in functioning, hospital (re)admission, placement in nursing homes and mortality [5–8]. Therefore, it is important to assess the needs of older adults with joint pain and comorbidity [9, 10].

The Camberwell Assessment of Need for the Elderly (CANE) is a structured interview that was developed to identify needs in 24 patient-related care items [11]. Until now, the CANE instrument was mostly used in populations with dementia or depression [e.g. 12, 13]. Very few studies explored care needs in an older primary care population [9, 14]. The most frequently identified unmet needs in these studies were found for the CANE items ‘visual/hearing impairment’, ‘information’, ‘physical illness’, ‘mobility/falls’ and ‘incontinence’ [9, 14]. Furthermore, a more recently published study in frail older adults found most unmet needs for the items ‘company’, ‘daytime activities’, ‘information’, ‘accommodation’ and ‘caring for another’ [15]. However, it is not known whether these results are applicable to a more specific older population with joint pain and comorbidity. In addition, little is known about factors that are associated with self-perceived needs, whilst one can imagine that many of the sociodemographic, physical and psychosocial factors that have been previously linked to levels of pain and disability [4], also influence care needs in older adults.

This study examined the self-perceived care needs in a primary care sample of older adults with joint pain and comorbidity. We identified the most prevalent needs and assessed associated factors.

## Methods

### Design and study population

This is a cross-sectional study using baseline data from a cohort study of older adults in the Netherlands with joint pain and comorbidity. In this study, data were collected by means of a postal questionnaire and a home visit with several physical tests and a face-to-face interview, including the structured CANE interview [16]. Participants were recruited from 22 general practices (GPs) in the region of Amsterdam and were eligible for participation if they were aged 65 years or over, had 2 or more chronic diseases registered in the electronic medical records of the GPs, and reported joint pain on most days in the past month (questionnaire) in at least one of eight joint pain sites: neck, back, shoulder, elbow, wrist/hand, hip, knee and ankle/foot. Details about the study design and recruitment process have been previously described [16]. In summary, almost 800 participants were eligible for participation, of which eventually 407 participants were included in the study. Within the group of eligible patients, we found no differences between participants and non-participants in age and gender. However, the non-participants had fewer chronic diseases, reported fewer joint pain sites and less pain. Data were collected between November 2010 and May 2013. The Medical Ethics Committee of the VU University Medical Center Amsterdam approved the study protocol. Written informed consent was obtained from all participants.

### Measurements and procedures

#### Outcome

Self-perceived care needs were identified using the CANE. This is a structured, multi-dimensional needs assessment that covers the environmental, physical and psychosocial domain, and identifies both met and unmet care needs [11]. While the CANE was originally developed to identify needs in 24 care items for use in old-age psychiatry, Walters et al. [17] showed that 11 items are not appropriate for a more general older population. Therefore, we decided to assess only the remaining 13 items: accomodation, household activities, food, caring for another, physical illness, medication use, sensory impairment, mobility/falls, self-care, memory, company, daytime activities and information. The CANE items show good validity and reliability in older populations with dementia [18]. Detailed and hierarchical questions are asked to identify problems, the nature and the severity of the problem and the extent to which help is received, for all 13 items. For each item, the questionnaire consists of five parts, to assess whether there is currently a need for that specific item (part 1), to determine whether there is informal assistance from family, friends and neighbours for this specific need (part 2), to determine whether the older person receives any help from local services to help with the problem (part 3), to determine whether the older person receives the right type of help with the problem (part 4), and to measure whether the older person is satisfied with the help received (part 5).

In this study, a met need was defined as receiving sufficient help to solve or reduce the problem, whereas an unmet need was defined as lack of help or insufficient help to reduce or solve the problem. The percentages of needs can be assessed on individual care item level, but also on domain level as previously shown by Field et al. [19]. Three domains can be distinguished: environmental needs, physical needs and psychosocial needs. Three trained interviewers, including the first author, conducted the CANE interviews.

#### Covariates

Sociodemographic covariates included age, gender, education (primary, secondary, college/university) and living situation (alone, not alone). Physical factors included pain intensity, number of chronic diseases, frailty and physical functioning. Pain intensity was measured with three items of the Chronic Pain Grade (CPG, score range 0–100, higher score indicates more pain) [20]. The presence of chronic diseases was derived from electronic medical records (score range 2–19; dichotomized to 2 versus ≥3) [16]. Frailty was considered to be present if participants met three or more of the five frailty phenotype criteria: weight loss, weakness, slowness, exhaustion, and low activity [21–23]. Physical functioning was assessed by the 10-item physical functioning subscale of the RAND-36 (score range 0–100; higher score reflects better physical functioning) [24]. Psychosocial factors included anxiety/depressive symptoms and social support. Anxiety and depressive symptoms were measured using the 14-item Hospital Anxiety and Depression Scale (HADS, score range 0–21, higher score indicates more symptoms) [25]. Social support was determined by the 12-item Social Support Scale (SSS, score range 12–60, higher score indicates less perceived social support) [26].

### Analyses

We used descriptive statistics to describe the study population and the proportion of care needs on care item level and domain level. Next, linear regression analysis was applied for physical needs, since this was the only outcome measure that was normally distributed. Since the needs in the other two domains and unmet needs in all three domains were not normally distributed, we dichotomised these outcomes by distinguishing participants with one or more needs from those without needs in the particular care domain. For the unmet needs, we distinguished participants with one or more unmet needs from those without unmet needs. The dichotomized outcomes were analysed with logistic regression analyses. Univariate associations between the selected variables and six outcomes were tested (data not shown). The variables with *p* < 0.10 on the outcomes were included in the multivariable models. We present the standardized regression coefficients (beta) or odds ratios (OR) with 95% confidence intervals (95% CI). In case of collinearity (spearman *r* >0.80), we included only the determinant with the strongest association in the model. Data were analyzed using SPSS version 20.0.

## Results

The baseline characteristics of the 407 participants are outlined in Table 1. Our sample was on average 76.8 years old (range 65.2–92.8) and about 62% was female. About 60% of the participants lived together with someone else, mostly their partner. The mean number of joint pain sites was 4.0 (SD 1.9); worse pain was most often reported in the back (27%), knee (18%), hand/wrist (15%) or hip (13%). Furthermore, almost half had more than two chronic diseases. The top ten of comorbid conditions included ischemic heart disease (62%), diabetes (37%), asthma or COPD (28%), cerebrovascular disease (24%), hearing problems (20%), malignancies (15%), chronic thyroid disorder (13%), peripheral arterial heart disease (12%), dizziness (9%) and visual problems (8%).

**Table 1:** Baseline characteristics of the study population (*n* = 407)

Gender, female *n* (%)	254 (62.4)	
Age, mean (SD)	76.8 (6.3)	
Living situation, alone *n* (%)	165 (40.5)	
Education, *n* (%)		
Primary	121 (29.7)	
Secondary	199 (48.9)	
College/university	87 (21.4)	
Pain intensity (0–100), mean (SD)	64.4 (17.3)	
Chronic diseases; ≥3 *n* (%)	197 (48.4)	
Frailty, yes *n* (%)	75 (18.4)	
Physical functioning (0–100), mean (SD)	48.7 (25.8)	
Anxiety symptoms (0–21), mean (SD)	5.1 (3.7)	
Depressive symptoms (0–21), mean (SD)	5.4 (3.5)	
Social support (12–60), mean (SD)	19.1 (8.4)	

CANE	Mean (SD)	≥1 needs *n* (%)
Total needs (0–13)	4.0 (2.1)	393 (98.3)
Total unmet needs (0–13)	0.3 (0.8)	88 (22.0)
Environmental needs (0–4)	1.2 (1.0)	277 (69.3)
Environmental unmet needs (0–4)	0.10 (0.33)	34 (8.5)
Physical needs (0–5)	2.5 (1.2)	381 (95.3)
Physical unmet needs (0–5)	0.14 (0.39)	49 (12.3)
Psychosocial needs (0–4)	0.3 (0.7)	90 (22.5)
Psychosocial unmet needs (0–4)	0.11 (0.41)	32 (8.0)

Our sample reported on average 4.0 care needs (SD 2.1), mostly physical needs (mean 2.5, SD 1.2) and to a lesser extent environmental needs (mean 1.2, SD 1.0) and psychosocial needs (mean 0.3, SD 0.7) (Table 1). Looking at care item level, most needs were identified for ‘physical illness’ (91%), ‘household activities’ (61%), ‘mobility/falls’ (53%) and ‘visual/hearing impairment’ (44%) (Table 2). Further evaluation of these care needs showed that the vast majority of the care needs were met. However, this was not the case for all items. Almost one quarter of our sample had at least one unmet need in the 13 care items, of which most unmet needs were identified in the psychosocial and environmental domains, especially regarding ‘company’ (67%), ‘daytime activities’ (37%), ‘caring for another’ (31%), ‘information’ (26%) and ‘accommodation’ (18%) (Table 2). Although only few people had needs regarding these care items, a large proportion was unmet.

**Table 2:** Descriptives of total needs, met needs and unmet needs for all 13 CANE items

	Total needs	Met needs^a^	Unmet needs^a^
	*N* (%)	*N* (%)	*N* (%)
Environmental needs			
Accomodation	78 (19.5)	64 (82.1)	14 (17.9)
Household activities	243 (60.8)	232 (95.5)	11 (4.5)
Food	126 (31.5)	122 (96.8)	4 (3.2)
Caring for another	29 (7.3)	20 (69.0)	9 (31.0)
Physical needs			
Physical illness	364 (91.0)	351 (96.4)	13 (3.6)
Medication use	146 (36.6)	143 (97.9)	3 (2.1)
Visual/hearing impairment	176 (44.0)	160 (90.9)	16 (9.1)
Mobility/falls	212 (53.0)	189 (89.2)	23 (10.8)
Self-care	91 (22.8)	91 (100)	0
Psychosocial needs			
Memory	36 (9.0)	31 (86.1)	5 (13.9)
Company	30 (7.5)	10 (33.3)	20 (66.7)
Daytime activities	27 (6.8)	17 (63.0)	10 (37.0)
Information	38 (9.5)	26 (74.3)	9 (25.7)

^a^% of met and unmet needs are based on the numbers of the total needs

Univariate associations were most pronounced between care needs and frailty, physical functioning, depression and social support and between unmet care needs and frailty, physical functioning and depression (data not shown). The other factors showed more variety in associations. The results of the multivariable analyses are presented in Table 3. Participants who were older, female, frail or had poor physical functioning reported more environmental needs. Higher age and poor physical functioning were also related to more physical needs, and additionally more chronic diseases seemed to be an important covariate. In the psychosocial domain, it were not so much the sociodemographic factors, but being frail, having depressive symptoms and perceiving less social support that were related to more psychosocial needs.

**Table 3:** Results of multivariable regression analyses of sociodemographic, physical and psychosocial factors associated with needs and unmet needs on care domain level^a^

	Environmental needs (0 vs. ≥1)	Physical needs (0–5)	Psychosocial needs (0 vs. ≥1)	Environmental unmet needs (0 vs. ≥1)	Physical unmet needs (0 vs. ≥1)	Psychosocial unmet needs (0 vs. ≥1)
	OR (95% CI)	Beta (95% CI)	OR (95% CI)	OR (95% CI)	OR (95% CI)	OR (95% CI)
Age (years)	1.07 (1.02–1.13)**	0.02 (0.00; 0.04)*				
Gender (female)	5.23 (2.87–9.53)***	0.06 (−0.15; 0.28)				2.64 (0.83–8.43)
Living situation (alone)						1.62 (1.13–2.31)**
Education: primary						
Secondary	1.44 (0.71–2.92)					
College/university	0.84 (0.37–1.89)					
Pain intensity (0–100)	1.00 (0.99–1.02)	0.00 (−0.01; 0.01)			1.01 (0.99–1.03)	
Chronic diseases (≥3)		0.35 (0.15; −0.56)**				
Frailty (yes)	4.36 (1.17–16.26)*	−0.11 (−0.41; 0.19)	2.40 (1.25–4.61)**	1.15 (0.48–2.75)	1.24 (0.59–2.62)	2.37 (0.86–6.51)
Physical functioning (0–100)^a^	0.95 (0.93–0.96)***	−0.02 (−0.02; −0.02)***	1.01 (0.99–1.02)	0.96 (0.95–0.98)***	0.97 (0.96–0.99)***	1.01 (0.99–1.03)
Anxiety symptoms (0–21)				1.10 (0.99–1.22)		1.02 (0.91–1.14)
Depressive symptoms (0–21)	1.00 (0.91–1.09)	0.00 (−0.03; 0.03)	1.17 (1.07–1.26)***	1.05 (0.92–1.19)	1.03 (0.93–1.14)	1.09 (0.94–1.26)
Social support (12–60)^b^			1.05 (1.01–1.08)**	1.02 (0.97–1.06)	1.03 (0.99–1.06)	1.07 (1.02–1.12)**

Only variables with *p* < 0.10 (univariate associations) on the outcomes were included in the multivariable models

*OR* = odds ratio, *95% CI* = 95% confidence interval, *Beta* = standardized regression coefficient

*P < 0.05, **P < 0.01, ***P < 0.001

^a^Higher score means better physical functioning

^b^Higher score means less social support

Looking at unmet needs, poor physical functioning was related to both more environmental and physical unmet needs, whereas less perceived social support and additionally living alone were related to more unmet psychosocial needs.

Overall, the sociodemographic and physical factors seemed mainly related to environmental and physical needs, whereas the psychosocial factors were more strongly related to psychosocial needs.

## Discussion

In this study, we explored self-perceived care needs and factors that are related to identified needs in older adults with joint pain and comorbidity. Our sample reported an average of 4.0 needs on 13 care items, mostly in the physical and environmental domains. The multivariable models showed that participants who were older, female, frail, had more chronic diseases or poor physical functioning reported more environmental and physical needs, of which especially poor physical functioning was related to more unmet needs. However, most needs in these domains were met. In contrast, although only few people reported psychosocial needs, a large proportion of these needs were unmet. It were especially the participants who lived alone, perceived less social support and reported more depressive symptoms that had more psychosocial needs.

Our sample of older adults with joint pain and comorbidity reported most needs in the care items ‘physical illness’, ‘household activities’ and ‘mobility/falls’, but most of these needs were met. We were able to compare the proportion of identified needs in our sample with a more general population [17] and a frail older population [15]. As compared to our study, the general population had more unmet needs in the physical domain, but less unmet needs in the psychosocial domain, whereas the frail population had higher unmet needs on almost all individual care items. The most obvious difference, compared to both mentioned studies was the relatively high percentage of needs in the item ‘accommodation’ in our sample. It is well known that most older adults prefer to stay in their own home as long as possible. This preference is captured in the term ‘ageing in place’ [27]. Maybe there is a high threshold to look for an alternative accommodation in our sample of older people with joint pain and comorbidity. Pain complaints are present, but may be perceived in this group as too mild to actually undertake actions.

Although our sample reported high numbers of needs in the environmental and physical domains, most of these needs were met. Some explanations come to mind. First, the Dutch health care system already has several facilities that arrange help when older adults become disabled and need help, in terms of appropriate treatment, medication, living adjustments and social services. Maybe the low levels of unmet needs in both domains just confirm that we have an adequate health care system in the Netherlands. Second, older adults with more health problems may become better in finding solutions themselves and being assertive in asking for help in their close environment. Third, many participants expressed a need on the item ‘physical illness’ because of their daily pain, but in many cases these needs were met. This may be due to appropriate treatment strategies, but could also be explained by the observation that many older people attribute pain and related problems to the normal process of aging. They accept the situation and learn to live and deal with the circumstances [28, 29].

Despite the low numbers of needs in the psychosocial domain, the multivariable regression analyses showed that especially living alone and perceiving less social support were associated with more unmet psychosocial needs. It is well known that social networks decrease with age [30], as older adults retire from work, outlive friends and have children that grow up, start a family and move away. In addition, pain and comorbidity cause more health-related disabilities, which prevent people from social engagement. Smaller social networks, isolation and loneliness are all indicative of the presence of a psychosocial need, which subsequently can cause less perceived social support, as support is often obtained from social networks. As lack of social support has been associated with decreased quality of life, more mental health problems and poorer social well-being [31–33], it seems important to target the psychosocial needs in clinical practice, by paying attention to the patient’s networks and their social care use. In the Netherlands, there could be a role for general practitioners. While consultation is often requested for disease-specific purposes, the psychosocial unmet needs ask for a more pro-active approach to identify problems in the social area. This may ask for more integrated care and close collaboration between health services (e.g. general practitioners, specialists, nurses) and social services (e.g. personal care, domestic help, social workers, transportation), which subsequently helps to better address individual care needs. Besides the more pro-active role of health services and social services, it remains very important that older adults and their social network members also take their own responsibility in signalling and encountering problems, in terms of starting the dialogue with spouse/family, approaching clinicians for advice and support and active coping towards solutions (self-management) that, for example, increase participation in social activities.

This study has several strengths. The CANE results are based on structured interview data. This provides knowledge that is strong in terms of generalization, precision and control. Furthermore, we used validated instruments and included variables that assessed mental health status, i.e. anxiety and depression. It was already mentioned that these variables could be important when assessing health care needs [34], but most studies failed to include these factors.

There are also some limitations. Overall, the CANE provides information about the presence of specific unmet care needs, which is important to optimize care for this specific population. However, conducting a structured CANE interview is quite burdensome, which makes it difficult to use this instrument in daily clinical practice. The CANE was originally developed to measure care needs from the perspective of the patient, the informal care giver and clinicians. We only assessed patients’ self-perceived care needs. However, in practice estimations about presence or absence of needs are often made by the clinicians or informal caregiver and it would be interesting to know to what extent these assessments are in agreement with the patient-reported needs, because low agreement could indicate lack of awareness/knowledge of current needs of the patient. This study did not determine objective health care needs. Comparison between objective and subjective health care needs could provide more information about factors that contribute to possible differences (e.g. social support, patients’ preferences, etc.).

In conclusion, older adults with joint pain and comorbidity seemed to be capable to adapt to their physical and environmental problems. Unmet needs are especially present in the psychosocial domain. Specific attention targeted at these unmet needs, preferably in an integrated setting of primary care and social care services, may improve psychosocial well-being of older adults with joint pain and comorbidity.
